# Ancient mtDNA Analysis of Early 16^th^ Century Caribbean Cattle Provides Insight into Founding Populations of New World Creole Cattle Breeds

**DOI:** 10.1371/journal.pone.0069584

**Published:** 2013-07-24

**Authors:** Camilla F. Speller, David V. Burley, Robyn P. Woodward, Dongya Y. Yang

**Affiliations:** 1 Department of Archaeology, University of York, York, United Kingdom; 2 Department of Archaeology, Simon Fraser University, Burnaby, British Columbia, Canada; Natural History Museum of Denmark, Denmark

## Abstract

The Columbian Exchange resulted in a widespread movement of humans, plants and animals between the Old and New Worlds. The late 15^th^ to early 16^th^ century transfer of cattle from the Iberian Peninsula and Canary Islands to the Caribbean laid the foundation for the development of American creole cattle (*Bos taurus*) breeds. Genetic analyses of modern cattle from the Americas reveal a mixed ancestry of European, African and Indian origins. Recent debate in the genetic literature centers on the ‘African’ haplogroup T1 and its subhaplogroups, alternatively tying their origins to the initial Spanish herds, and/or from subsequent movements of taurine cattle through the African slave trade. We examine this problem through ancient DNA analysis of early 16^th^ century cattle bone from Sevilla la Nueva, the first Spanish colony in Jamaica. In spite of poor DNA preservation, both T3 and T1 haplogroups were identified in the cattle remains, confirming the presence of T1 in the earliest Spanish herds. The absence, however, of “African-derived American” haplotypes (AA/T1c1a1) in the Sevilla la Nueva sample, leaves open the origins of this sub-haplogroup in contemporary Caribbean cattle.

## Introduction

Initiated in 1492 by the voyage of Christopher Columbus, the Columbian Exchange involved a dramatic and widespread transfer of people, animals, plants, diseases, and ideas between the New and Old Worlds [1], [2]. Iberian animal domesticates, including cattle, were introduced successfully to the Caribbean throughout the late 15^th^ and early 16^th^ centuries. The ability of these animals to survive and potentially prosper within this tropical landscape was essential for long-term success in Spanish settlement and expansion into the Americas [2], [3]. In Iberia, cattle production for meat and hides was a substantive component of economy, especially focused in the southern territory of Andalusia from which New World colonization efforts were staged [4]. New World cattle imports also came from the Canary Islands, a Spanish colony since 1479, and a mid-way stopping point for Spanish ships destined for the Caribbean [5].

Rouse [5] estimates Spanish cattle imports to the Caribbean between 1494 and 1512 were less than 1,000 head and probably no more a few hundred. Historical accounts indicate these herds were relatively adaptable, and they proliferated across the Caribbean landscape [6]. On Hispaniola (Haiti/Dominican Republic) alone, free ranging cattle herds numbered in the thousands of animals in the early to mid 16^th^ century, and the export of hides was a principal economic activity [7]. These historic observations are consistent with archaeological deposits dominated by cattle remains in early Spanish colonizing sites [8]. It was this core ancestral stock that was subsequently imported to both North and South America during the Spanish conquest era beginning in 1512, laying the foundation for the American creole breeds [5]. A second wave of cattle introductions began a century later, predominantly associated with the trans-Atlantic slave trade [9]–[11]. Between 1595 and 1880, over 26,000 voyages brought slaves, live animals and food crops from Africa to the Americas, including the Caribbean [9], [11]–[13]. Among these imports were cattle from northern and continental Europe, Africa, the Atlantic islands and India, thereby adding to the genetic diversity of New World creole populations [5], [9], [10], [14]–[17].

One cannot question the ancestral role of Iberian cattle for New World creole populations. There is, however, considerable debate as to the genetic composition of the original stock, particularly as it relates to African contributions [15], [18]–[22]. Analyses of *Bos taurus* mtDNA control-region sequences previously defined three predominant and geographically structured taurine haplogroups [23]: T1 dominantly occurs in Africa; T2 originates in the Near East and Western Asia; and T3 is found throughout Europe [20], [23], [24]. Although whole genome analyses reveal a far more complex view of cattle domestication [25], [26], the geographic patterns associating T3 predominantly with Europe, and T1 and its subhaplogroups with Africa, still conforms to the original pattern [18], [25], [27], [28].

Contemporary American creole breeds are of both European (T3) and African (T1) haplogroups, implying dual geographic origins [15], [19]–[21], [29]–[31]. Haplogroup T3, the one most prevalent in European taurine cattle, is the most common in American creole breeds, ranging from 47–72% [20], [29], [31]. Many of the T3 haplotypes found in creole cattle are similar to those identified in cattle from the Iberian Peninsula and Canary Islands, a finding consistent with known migration routes for cattle into the New World [15], [20]. Haplogroup T1, which is characteristic of almost all sub-Saharan and West African cattle, is found in modern Iberian cattle and American creole breeds at lower frequencies [20], [29], [31]. In particular, the origins of the T1 sub-haplogroup AA (“African-derived American”) [32], more recently designated T1c1a1 [18], has been contentious [20], [21], [29], [31], [32], explained either by founder effect from the earliest Iberian cattle [33] and/or as an introduction through later crossbreeding of African cattle, perhaps in association with the African slave trade [21].

**Figure 1:**
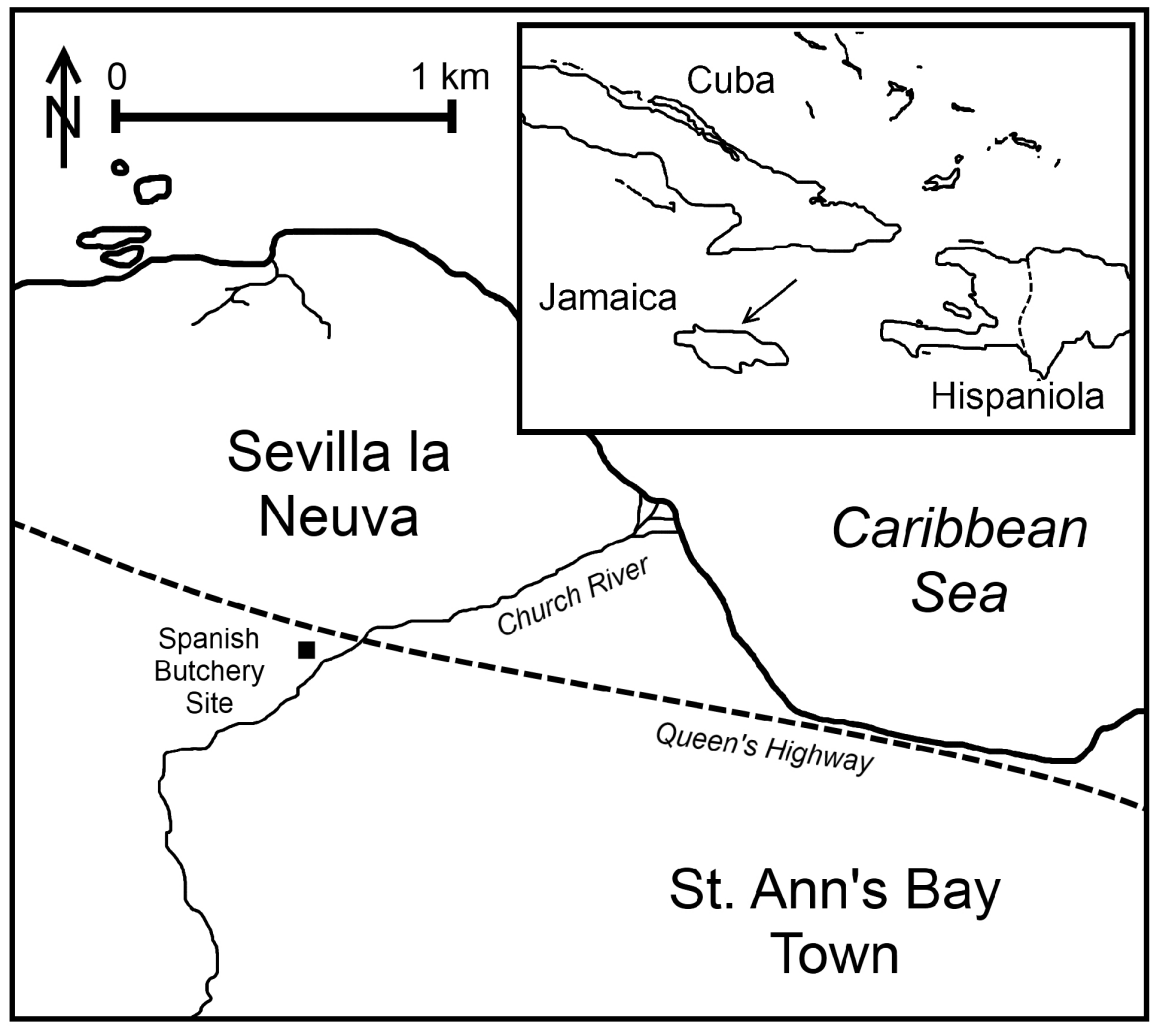
Location of the 1509–1534 Spanish Colony of Sevilla la Nueva, Jamaica. The darkened square is the butchery site from which ancient DNA bone samples were excavated.

Genetic research into American cattle and the preceding debate focus solely on DNA composition within contemporary populations, leaving the origin and timing of T1 and its subhaplogroups in New World creole cattle as unresolved issues. Here we present an ancient DNA analysis of early 16^th^ century cattle remains from a butchery site at Sevilla la Nueva, the first Spanish colony in Jamaica (Figure 1). The colony was founded in 1509 on St Ann’s Bay under the orders of Diego Colon, Governor of the Indies and son of Christopher Columbus. Ultimately abandoned in 1534, its initial purpose was as a centre for agricultural and livestock production in support of other colonizing ventures into Central and South America [34], [35]. Sevilla la Nueva cattle were brought originally from herds on Hispaniola, and they provided breeding stock to the America’s through transfer in Panama [5]; as such, this site presents an ideal context for testing hypotheses into the founding populations of creole cattle. Meat processing at the butchery site, however, was focused dominantly on sheep with only small numbers of identifiable cattle bone present within the excavated assemblage (Text S1). The sample for ancient DNA analysis accordingly is limited, including 24 archaeological specimens.

### Sample Context

**Figure 2:**
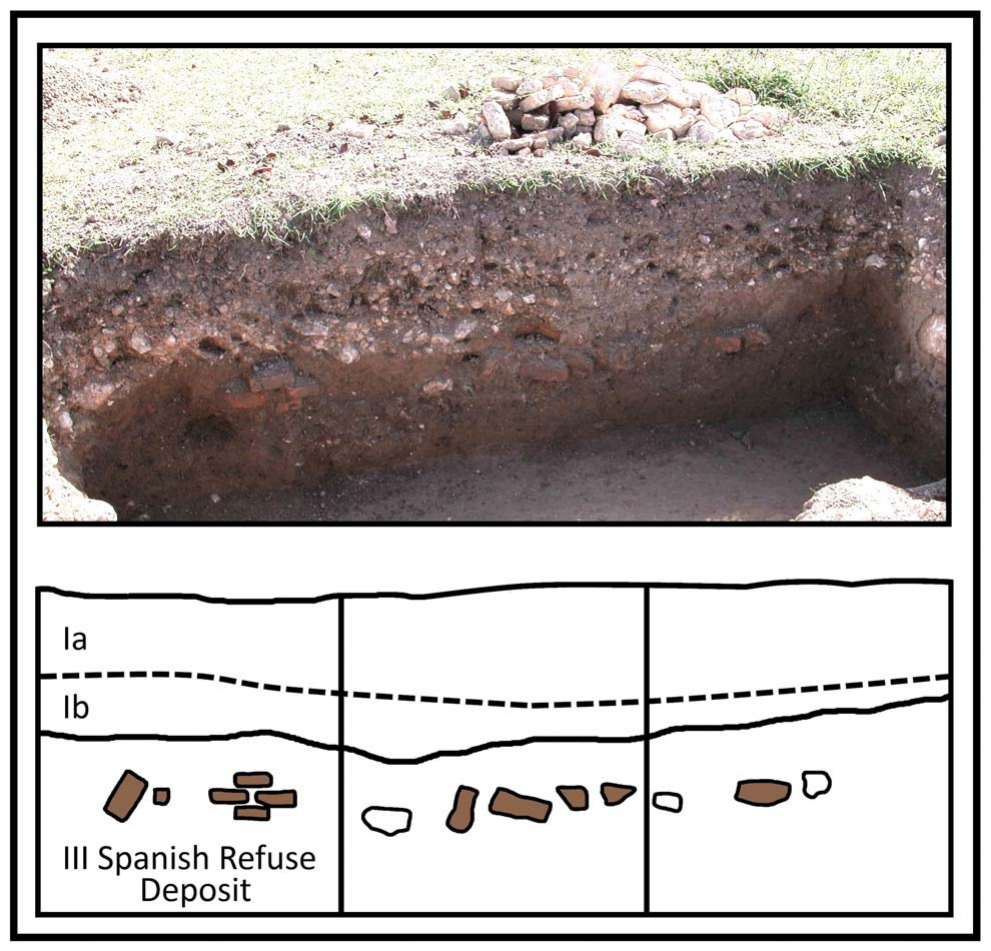
Stratigraphic profile of south face Unit 3, 2004 excavation. Stratum I is compacted alluvial gravel that required the use of a pick-axe for removal. The dotted line separating substrata Ia from Ib is the break between gravels without cultural material (Ib) and the upper agricultural zone with scattered artifacts from the British and modern eras (Ia). Stratum II is a fine alluvial silt that is deposited over the Spanish occupation floor in other areas of the site. Stratum III is the Spanish occupation and refuse midden from which bone samples were acquired. The bricks (darkened) and rocks (open) are not associated with architectural features but are part of the last deposit of refuse fill by the Spanish. The identified T1 haplogroup sample was recovered from Unit 3-E, Stratum III immediately to the north of this profile.

Because the sample size is small, the integrity of sample context and its clear association with the 1509–1534 period of Spanish occupation at Sevilla la Nueva is essential. The butchery site was positioned along the shore of a now in-filled stream branching off from the Church River (Text S1). Concentrated refuse from the Spanish operation, including butchered bone, Spanish ceramic wares, indigenous Taino ceramic wares and a limited assemblage of other artifacts was variably deposited across the site. After the butchery ceased to operate, periodic flooding of the river resulted in deposition of silts and gravels. The flood events cumulatively capped the Spanish occupation with a compact and almost impenetrable layer of alluvial material up to 40 cm thick (Figure 2). That this layer sealed the Spanish archaeological remains from later disturbance is illustrated by the exclusive nature of Spanish ceramic types occurring in deposits below it; all have date ranges consistent with the 1509–1534 AD period of settlement for Sevilla la Nueva (Table S1). Each of the cattle bone samples selected for DNA analysis was excavated from deposits beneath this gravel (Stratum III), in stratigraphic association with the ceramics (Table 1). Therefore, the samples selected for ancient DNA analysis represent ancestral Caribbean stock and they potentially provide insight into the origins of the T1 haplogroup for creole cattle of the Americas.

**Table 1:** Provenience information and results of mtDNA D-loop amplifications of Sevilla la Nueva cattle samples.

Specimen number (Lab Code)	Element	Provenience	Lab	Sequence Read	GenBank Accession (Haplogroup)
SN-Sta32-5:F1 (JC1)	Innominate	Stratum III, Unit 3-E	SFU, UC	16029–16147	KF152925 (T1)
SN-Sta32-5:F2 (JC2)	Patella	Stratum III, Feature Layer 2	SFU	Fail	–
SN-Sta32-5:F3 (JC3)	Astragalus	Stratum III, Unit 3-A	SFU, UC	16029–16135	KF152926 (T3)
SN-Sta32-5:F4 (JC4)	Radius (immature)	Stratum III, Unit 10-NW	SFU, UC	16029–16135	KF152927 (T3)
SN-Sta32-5:F5 (JC5)	Long bone shaft (immature)	Stratum III, Feature Layer 2	SFU	Fail	–
SN-Sta32-5:F6 (JC6)	Tooth	Stratum III, Unit 3-H	SFU	16029–16147	KF152928 (T3)
SN-Sta32-5:F7 (JC7)	Femur	Stratum III, Unit 3-G	SFU	Fail	–
SN-Sta32-5:F8 (JC8)	Vertebra	Stratum III, Unit 15	UC	Fail	–
SN-Sta32-5:F9 (JC9)	Astragalus	Stratum III, Unit 3-I	SFU	Fail	–
SN-Sta32-5:F10 (JC10)	Long bone shaft	Stratum III, Unit 10-NW	SFU	Fail	–
SN-Sta32-5:F11 (JC11)	Long bone shaft	Stratum III, Unit 14	UC	16029–16147	KF152929 (T3)
SN-Sta32-5:F12 (JC12)	Tooth	Stratum III, Unit 13	UC	Fail	–
SN-Sta32-5:F13 (JC13)	Astragalus	Stratum III, Unit 3-D	SFU	16029–16147	KF152930 (T3)
SN-Sta32-5:F14 (JC14)	Tooth	Stratum IIISB, Unit 3-H	UC	Fail	–
SN-Sta32-5:F15 (JU1)	Rib	Stratum III, Unit 14	UC	Fail	–
SN-Sta32-5:F16 (JU2)	Long bone shaft	Stratum III, Unit 6-NE	UC	16029–16147	KF152931 (T3)
SN-Sta32-5:F17 (JU3)	Long bone shaft	Stratum III, Unit 3	UC	Fail	–
SN-Sta32-5:F18 (JU4)	Rib-vertebra	Stratum IIISB, Unit 3	UC	Fail	–
SN-Sta32-5:F19 (JU5)	Long bone shaft	Stratum III, Unit 3-G	UC	Fail	–
SN-Sta32-5:F20 (JU6)	Long bone shaft	Stratum III, Unit 10-SE	UC	Fail	–
SN-Sta32-5:F21 (JU7)	Rib-vertebra	Stratum IIISB, Unit 3-G	UC	Fail	–
SN-Sta32-5:F22 (JU8)	Radius (immature)	Stratum III, Unit 3-G	UC	Fail	–
SN-Sta32-5:F23 (JU9)	Rib-vertebra	Stratum III, Unit 3-E	UC	Fail	–
SN-Sta32-5:F24 (JU10)	Vertebral epiphysis	Stratum III, Feature Layer 2	UC	Fail	–

Note: Sequence reads indicate fragments lengths obtained after primer sequences had been removed; coordinates based on BRS [38].

## Results and Discussion

Mitochondrial DNA was successfully amplified from but seven of the 24 specimens in spite of the samples being only 475–500 years old (GenBank Accessions KF152925-KF152931). This low amplification rate is consistent with high rates of DNA degradation in warm, humid, equatorial climates such as Jamaica [36], [37]. The estimated thermal age for the bones is 12,750 years before present with a λ of 0.0273 [37]; this age predicts high DNA fragmentation and <10% of DNA fragments longer than 100bp. Successfully amplified samples display mitochondrial sequences consistent with taurine cattle (*Bos taurus)* rather than Zebu (*Bos indicus*).

Previously, taurine haplogroups were defined by relatively few control-region transitions compared to the bovine reference sequence (BRS) [38]: T defined by a transition at 16255; T1 by transitions at 16050 and 16113; T2 by 16185 and a G to C transversion at 16057; and T3 was identical to the BRS [23]. Bayesian analyses of ancient aurochsen, and modern and ancient cattle complete mtDNA genomes indicate that classic haplogroups definitions are simplistic with at least some of the T3 haplotypes grouping in polyphyletic clades [26]. Additional full genome sequencing redefines the predominant macro-haplogoup T, and identifies additional rare haplogroups P, Q and R [25]. Haplogroup T is now separated into sister clades of T1′2′3 and T5, the former encompassing the previously defined haplogroups of T1, T2, and T3 (as well as T4, which clusters within T3). While the majority of the newly defined sub-haplogroups under T1, T2 and T3 are defined by mtDNA coding regions polymorphisms [18], [25], one exception is the “African-derived American” haplogroup AA/T1c1a1 which can still be distinguished by four control-region polymorphism (16053–16122–16139–16196) [18]. In this study, six samples displayed *Bos taurus* sequences matching the BRS (T3); the seventh sample displayed the classic T1 motif of 16050 and 16113 (Table 1).

Ancient DNA analyses of Sevilla la Nueva samples provide a limited data set for interpretation, and it would be inappropriate to overstate the results with such low numbers. Nevertheless, the secure nature of sequenced specimens in early 16^th^ century archaeological deposits associates these samples with ancestral cattle herds in the Caribbean. The Sevilla la Nueva data without doubt document nodal T3 and T1 haplotypes in New World cattle herds prior to the widespread African slave trade in the Americas. While only one T1 haplotype was identified at Sevilla la Nueva, it still implies that the African genetic contribution to Iberian cattle came about early, presumably relating to earlier cattle imports into the Iberian Peninsula from North Africa by the Moors [22], [33], [39], [40]. Additional T1 contributions to New World cattle from the African slave trade or from more recent introductions cannot be ruled out, but neither can serve as the sole explanation for the presence of the T1 haplogroup in contemporary American cattle.

The absence of sub-haplogroup AA/T1c1a1 in the Sevilla la Nueva cattle remains is notable, despite the small sample size. Analyses of contemporary cattle from Antigua, St. Lucia, and Guadeloupe have relatively high frequencies of both T3 and AA/T1c1a1 (the latter ranging as high as 39%), and with low frequencies of the nodal T1 type [21], [31]. Previously, the detection of AA/T1c1a1 in Caribbean, Spanish Retinta [31] and Lidia cattle [41], and its apparent absence in African cattle, suggested an origin in 16^th^ century Spanish herds. Based on the patchy distribution of AA/T1c1a1 in the Americas, and supported by the recent detection of AA/T1c1a1 in Egyptian cattle [18], others have proposed a direct introduction from Africa at a later date [20], [29]. In this latter scenario, recent backcrossing of Brazilian Retinta cattle with Iberian breeds in the 1960s and 1970s could account for its distribution in contemporary Iberian cattle [39], [40]. The absence of AA/T1c1a1 both in Bronze age Iberian cattle [33] as well as at Sevilla la Nueva, leaves open the question of AA/T1c1a1 origins. The presence of AA/T1c1a1 in the Caribbean may have occurred early via Spanish imports procured from the Canary Islands [14]. However, a later introduction is more probable. Frequencies of AA/T1c1a1 are particularly high on Antigua, Guadeloupe, and St. Lucia. These islands, however, were not settled until the 17^th^ century, when English, French and Dutch colonist replaced indigenous Carib peoples. Thus, T1 sub-haplogroup may have been introduced by European settlers during this later colonization of the Windward and Leeward islands. Further ancient DNA analysis of ancient Iberian, Caribbean and American continental cattle dating pre- and post-1600AD will be required to clarify the timing and origins of the so-called “African-derived American” haplotype in the New World.

Over the last decade, nuclear and mtDNA analyses of cattle have provided an abundance of data on contemporary creole cattle breeds, raising a number of hypothesis that can only be tested with historic data. Other studies in both the Old and New Worlds have illustrated how ancient DNA analysis can uncover historic patterns of domestic animal movements that have been masked by subsequent translocations and modern breeding programs [42], [43]. This study provides the foundation for a time-series analysis of archaeological samples from other Caribbean and mainland archaeological sites to more clearly reveal the historical and relative contributions of African and Iberian cattle to creole stocks, and clarify insight into the development of both New and Old World cattle breeds.

## Materials and Methods

### Ethics Statement

Archaeological research at Sevilla la Nueva is conducted under Jamaica National Heritage Research Permits and Agreements to RPW dated 3 October 2004 and 15 January 2009. All necessary permits were obtained for the described study, which complied with all relevant regulations.

### Samples and Context

The 24 ancient cattle bone samples were excavated from the archaeological site of Sevilla la Nueva, the first Spanish colony on Jamaica (Table 1). The bone samples for ancient DNA analysis were identified as cow or more generally as large bovid in the Simon Fraser University zooarchaeological laboratory using comparative morphological traits. Following analysis, the repository for the bones samples will be the Jamaica National Heritage Trust, Kingston, Jamaica.

Archaeological research at Sevilla la Nueva has occurred intermittently over the past 75 years [44]. Burley and Woodward recovered the 24 ancient DNA samples from excavations of a Spanish butchery and meat-processing site undertaken in 2004 and 2009. The samples come from refuse midden in which abundant butchered bone and other materials are present. Compact alluvial gravel originating from a tributary of the nearby Church River stratigraphically caps architectural features of the butchery as also the refuse deposits (Figure 2). Thus, the 24 bone samples are sealed within the Spanish occupation floor, having a maximum date of 1534. Additional details for the Sevilla la Nueva site and the excavation project are provided as supplemental information (Text S1).

### DNA Extraction and mtDNA Analysis

Bone samples were prepared and extracted in the ancient DNA laboratory in the Department of Archaeology, Simon Fraser University (SFU) as well as in the ancient DNA laboratory in the Department of Archaeology, University of Calgary (UC). DNA was extracted following a modified silica spin column protocol [45], [46]. Standardized preparation and extraction protocols were maintained between laboratories as outlined in supplemental information (Text S1). PCR amplifications targeted the mtDNA control-region distinctive of taurine haplogroups [46] (Text S1). Sequences were edited visually with primer sequences removed and base pair ambiguities examined using ChromasPro software (www.technelysium.com.au). All returned sequences were entered into GenBank through the BLAST application (http://www.ncbi.nlm.nih.gov/BLAST/) to ensure they did not match unexpected species or sequences. Multiple alignments of the obtained sequences and previously published mtDNA reference sequences were conducted using Clustal W [47] through BioEdit [48]. *B. taurus* haplogroups were assigned based on the mutations defined in Troy et al. [23] and Bonfiglio et al. [18]. Criteria used to ascertain the authenticity of the sequences are presented in Text S1.

## Supporting Information

Table S1 **Spanish ceramic wares recovered from Stratum III context in association with analysed cow bone samples at Sevilla la Nueva.** Manufacturing date ranges are taken from the on-line digital type collection at the Florida Museum of Natural History <www.flmnh.ufl.edu/histarch/gallery_types/type_list.asp>.
(DOCX)

Text S1 **Additional Information on historical and archaeological context and ancient DNA analysis.**
(DOCX)
