# Supplementary material for: Ancient mtDNA Analysis of Early 16th Century Caribbean Cattle Provides Insight into Founding Populations of New World Creole Cattle Breeds
Source: PLoS One. 2013 Jul 24;8(7):e69584. doi: 10.1371/journal.pone.0069584 (PMC3722109; doi:10.1371/journal.pone.0069584)
Supplement: Table S1 — Spanish ceramic wares recovered from Stratum III context in association with analysed cow bone samples at Sevilla la Nueva. Manufacturing date ranges are taken from the on-line digital type collection at the Florida Museum of Natural History <www.flmnh.ufl.edu/histarch/gallery_types/type_list.asp>. (DOCX) [file pone.0069584.s001.docx]

**Table S1. Spanish ceramic wares recovered from Stratum III context in association with analysed cow bone samples at Sevilla la Nueva.** Manufacturing date ranges are taken from the on-line digital type collection at the Florida Museum of Natural History <www.flmnh.ufl.edu/histarch/gallery_types/type_list.asp>.

| **Ceramic Types** | **Ceramic Function** | **Date Range** | **Number of Identified Samples** |
| --- | --- | --- | --- |
| Cuenca Tile | Majolica Decorative Tile | 1500-1575 | 2 |
| Caparra Blue | Majolica Tableware | 1492-1600 | 3 |
| Columbia Plain | Majolica Tableware | 1490-1650 | 186 |
| Isabella Polychrome | Majolica Tableware | 1490-1580 | 2 |
| Yayal Blue on White | Majolica Tableware | 1490-1625 | 6 |
| Melado | Lead-glazed Tableware | 1490-1550 | 26 |
| Feldspar Inlaid | Unglazed Tableware | 1500-1600 | 2 |
| Bizcocho | Unglazed Tableware | 1500-1550 | 32 |
| Green Bacin/Lebrillo | Lead-glazed Utilitarian Ware | 1490-1600 | 12 |
| Early Olive Jar | Unglazed Utilitarian Ware | 1500-1570 | 262 |
| Lead-glazed Redware | Utilitarian Ware | 1500-1600 | 44 |
| Lead-glazed Earthenware | Utilitarian Ware | 1490-1900 | 9 |
