# Supplementary material for: Ancient mtDNA Analysis of Early 16th Century Caribbean Cattle Provides Insight into Founding Populations of New World Creole Cattle Breeds
Source: PLoS One. 2013 Jul 24;8(7):e69584. doi: 10.1371/journal.pone.0069584 (PMC3722109; doi:10.1371/journal.pone.0069584)
Supplement: Text S1 — Additional Information on historical and archaeological context and ancient DNA analysis. (DOCX) [file pone.0069584.s002.docx]

**S1 Text - Additional Information on Historical and Archaeological Context and Ancient DNA Analysis**

**Historical and Archaeological Context**

Sevilla la Nueva was established in 1509 on the delta fan of the Church River where braided distributary channels emptied into St. Anne’s Bay [[1](#_ENREF_1)]. Jamaica lacked exploitable gold deposits, leaving Sevilla la Nueva as a centre for agricultural and livestock production in support of Spanish conquest and colonization throughout the New World [[2](#_ENREF_2)]. Being Jamaica’s first capital, the settlement included a governor’s castle and defensive works, an abbey, an industrial complex as well as residences for its populace [[3](#_ENREF_3)]. The capital was transferred to Villa de la Vega (Spanish Town) on Jamaica’s south coast in 1534, and the colony was all but abandoned [[2](#_ENREF_2)]. In 1655, the British ended the Spanish era of Jamaican history, capturing and redistributing lands throughout the island. The Sevilla la Nueva site was incorporated into the sugar estate of Capt. Richard Hemmings in 1670 [[4](#_ENREF_4)]. The Jamaican government purchased the property in 1971, establishing the Seville Heritage Park for interpretation to the general public. This park was elevated to the tentative category for a UNESCO World Heritage Listing in 2009.

Modern era “rediscovery” of archaeological remains for Sevilla la Nueva occurred in 1937 [[3](#_ENREF_3)]. Several projects on different aspects of the site have been undertaken since that time. Discovery of the butchery occurred in 2004, when Woodward and Burley began systematic survey for Spanish residential features in the Heritage Park. The site was isolated from both the colony’s industrial area and its administrative core, positioned 250m inland and to the south of the latter. In 2004, a limited excavation of 27m^2^ exposed a scatter of brick and cobble features as well as associated refuse deposits of bone and other materials. Expansion of the excavation in 2009 provides clarification of the site as a butchery [[5](#_ENREF_5)]. From these two years of excavation 17,358 bone fragments have been recovered, most with butchering marks but not burning, as would be characteristic of household consumption. Of the 1,995 fragments that could be identified, cattle account for only 6.7% with sheep dominating the assemblage. This is surprising given historical accounts of cattle introductions to Jamaica and their economic importance [[2](#_ENREF_2)]. Sheep herding, however, is also well documented [[6](#_ENREF_6)].

The butchery site was strategically positioned on the eastern bank of a small branching stream from the Church River. This location proved consequential for preservation of Spanish archaeological deposits, as post-abandonment floods resulted in the deposition of a thick (up to 40 cm) compacted cover of alluvial silts and gravels across the site area (Figure 2). Deposition of this alluvial stratum occurred predominantly between 1534 and 1670, following the abandonment of Sevilla la Nueva by the Spanish but prior to the establishment of the Hemmings’ plantation. Scattered British plantation era and modern ceramics, as well as other artifacts are found only in the top 15 to 20 cm of this layer (Stratum Ia, Figure 2), this constituting the late 17^th^ through 20^th^ century agricultural zone. Below the gravel layer, ceramics are exclusively Spanish or they are of indigenous Taino origin. Ceramic types and date ranges associated with the former are consistent with the temporal interval during which the Sevilla la Nueva colony was occupied (1509-1534). The indigenous ceramics potentially reflects the Spanish system of *repartimiento,* where Taino men were forcibly employed as a source of labor [2]. All of the bone samples employed in the study occur in stratigraphic association (Stratum III, Figure 2) with the Spanish and Taino ceramics.

**Ancient DNA Extraction and Amplification**

Analyses in both the Simon Fraser University (SFU) and University of Calgary (UC) laboratories followed the same protocols. Approximately 0.5-1.5g of bone was sampled from each skeletal element. Prior to DNA extraction, bone samples were submerged in 6% sodium hypochlorite (100% commercial bleach) for 7 min to remove possible surface contamination, then rinsed twice in distilled water. The samples were UV irradiated in a cross-linker for 30 min on two sides, and left to dry before being crushed into powder. The resulting bone powder was incubated overnight in a lysis buffer (0.5 M EDTA pH 8.0; 0.25-0.5% SDS; 0.5 mg/mL proteinase K) in a rotating hybridization oven at 50°C. DNA extraction was performed using a modified silica-spin column protocol [[7](#_ENREF_7),[8](#_ENREF_8)]; 100 μL of DNA extract was eluted from each sample.

PCR amplifications were performed on an Mastercycler Personal or Mastercycler® ep (Eppendorf) Thermocycler using a 30 μL reaction volume containing 1.5X Applied Biosystems™ Buffer, 2 mM MgCl_2_ 0.2 mM dNTP, 1.0 mg/mL BSA, 0.3 μM each primer [[9](#_ENREF_9)], 3.0-4.0 μL DNA sample and 2.5-3.75 U AmpliTaq Gold (Life technologies Inc). PCRs were conducted for 60 cycles using an annealing temperature of 52-55°C. Five uL of PCR product from each sample were separated on a 2% agarose gel, and visualized using SYBR Green™ (Life Technologies Corporation, Carlsbad, CA). Successfully amplified samples were sequenced at the Central Facility of the Institute for Molecular Biology and Biotechnology Laboratory at McMaster University, at the Macrogen Ltd. sequencing facility in Seoul, Korea or Eurofins MWG Operon, using both forward and reverse primers.

## Contamination Controls and DNA authenticity criteria

Several measures were taken to decrease the likelihood of contamination and authenticate the obtained sequences. The extraction and amplification of the samples was conducted in dedicated ancient DNA laboratories which follow strict contamination control protocols, such as: the separation of the pre-PCR and post-PCR work spaces; the use of ancient DNA dedicated equipment including clothing, equipment and reagents. Multiple blank extracts and PCR negative controls were included in each batch of analyses – none of which yielded amplification bands of target length, though primer-dimers of less than 100bp were occasionally observed. Repeat extractions and/or repeat PCR amplifications were conducted for each sample to confirm that the obtained sequences were reproducible. Additionally, three samples underwent repeat DNA extractions and amplifications in two separate ancient DNA laboratories (SFU and UC, Table 1) to provide independent replications. For all successfully amplified samples, sequences obtained from repeat extracts and/or amplification were identical to the initial amplifications although one sequence (JC6) displayed a characteristic miscoding lesions caused by DNA degradation (C→T transition) [[10](#_ENREF_10),[11](#_ENREF_11)], which was verified through repeat amplifications and sequencing.

**References Cited**

1. Waters MR, Giardino JR, Ryter DW, Parrent JM (1993) Geoarchaeological investigations of St. Ann's Bay, Jamaica: The search for the Columbus Caravels and an assessment of 1000 years of human land use. Geoarchaeology 8: 259-279.

2. Padrón FM (2003) Spanish Jamaica (English Translation of Jamaica Española,1952). Kingston: Ian Randal Publishers.

3. Woodward RP (2009) Sevilla la Nueva: A review of 72 years of archaeological investigations. In: Tortello R, Greenland J, editors. Xaymaca Life in Spanish Jamaica 1494-1655. Kingston: The Institute of Jamaica. pp. 32-44.

4. Aarons GA (1983) Sevilla la Nueva: Microcosm of Spain in Jamaica, Part 1: The historical background. Jamaica Journal 16: 37-46.

5. Burley DV, Woodward R, Peters A, Waters G (2009) Archaeological Research at Sevilla Nueva, Jamaica’s First Spanish Capital. National Geographic Research and Exploration Committee (Grant 8522-08), on file with National Geographic Society and the Senior Author.

6. Wynter S (1983) Report on archival materials relating to Spanish Jamaica in Spain. Unpublished report on file with the Jamaica National Heritage Trust, Kingston.

7. Speller CF, Kemp BM, Wyatt SD, Monroe C, Lipe WD, et al. (2010) Ancient mitochondrial DNA analysis reveals complexity of indigenous North American turkey domestication. Proceedings of the National Academy of Sciences 107: 2807-2812.

8. Yang DY, Eng B, Waye JS, Dudar JC, Saunders SR (1998) Improved DNA extraction from ancient bones using silica-based spin columns. American Journal of Physical Anthropology 105: 539-543.

9. Yang DY, Liu L, Chen X, Speller CF (2008) Wild or domesticated: DNA analysis of ancient water buffalo remains from north China. Journal of Archaeological Science 35: 2778-2785.

10. Brotherton P, Endicott P, Sanchez JJ, Beaumont M, Barnett R, et al. (2007) Novel high-resolution characterization of ancient DNA reveals C > U-type base modification events as the sole cause of post mortem miscoding lesions. Nucleic Acids Research 35: 5717-5728.

11. Gilbert MTP, Binladen J, Miller W, Wiuf C, Willerslev E, et al. (2007) Recharacterization of ancient DNA miscoding lesions: insights in the era of sequencing-by-synthesis. Nucleic Acids Research 35: 1-10.
